# Editorial: Eating behavior and mental health during the COVID-19 pandemic

**DOI:** 10.3389/fnut.2023.1295557

**Published:** 2023-11-17

**Authors:** Rubia Carla Formighieri Giordani, Jonas Augusto Cardoso da Silveira, José Aparecido Da Silva

**Affiliations:** ^1^Department of Nutrition, Federal University of Paraná, Curitiba, Brazil; ^2^Postgraduate Program in Behavioral Sciences, University of Brasilia, Brasilia, Brazil

**Keywords:** eating behavior, habits and behaviors, pandemics, COVID-19, mental health

Eating behavior is a complex set of actions encompassing what, where, how and with whom we eat. It involves phylogenetic and ontogenetic factors, as well as the ability to control food intake through physiological mechanisms such as neurotransmitters, hormones, sensory receptors, and metabolism (1). External stimuli (such as the organoleptic properties of food), psychological pressure, and the social environment also affect food choices (2–4). Thus, eating behavior emerges from the interaction of the individual's biological characteristics with economic, social, and cultural contexts.

Beyond nourishing the body, food occupies a privileged place in human life in creating and maintaining social ties. During the COVID-19 pandemic, changes in social life due to city-wide movement restrictions and social distancing recommendations have altered food acquisition (Besoro-Moreno et al.) and eating patterns (Paz-Graniel et al.) and commensality (Di Nucci et al.). This is the scope of this Research Topic: the understanding of different aspects of food, nutrition, and eating behaviors associated with psychological factors linked to the pandemic (Wu et al.), brain metabolism (Juby et al.) and COVID-19, eating disorders (Yang et al.) as well as the changes imposed by the pandemic on lifestyle and consumption habits (Galvão et al.).

Preparing to manage a pandemic involves considerable time and planning. Based on the knowledge acquired from facing other severe epi or pandemics, four methods have been frequently used to manage and control the viral spread: (1) risk communication to raise public awareness, (2) vaccines and antiviral therapies, (3) adherence to preventive behaviors, and (4) restrictive measures on movement (5).

The effects of a long and uncertain pandemic have two major facets. On the one hand, it is known that the consequences of COVID-19, especially the damage caused directly by the virus infection, have led to various complications, or even neurological diseases resulting from neuroinflammation of the Central Nervous System (CNS) (Juby et al.). In addition to these, a multitude of cognitive and emotional dysfunctions, sensory ones such as the loss of smell and taste, and even problems regarding motor mobility and strokes. On the other hand, the stress associated with work, lockdowns, physical, and social isolation, as well as the long social distancing and quarantine, which are responses imposed by health and government authorities commonly used to contain the spread of the coronavirus (in this case, its SARS-CoV-2 version), also substantially affect human mental health. The combination of social isolation, information overload, interruptions to daily life, exacerbation of unemployment rates, and economic difficulties have caused growing feelings of anticipatory grief, worry, and anxiety around the world (5, 6).

All of this means that the pandemic, whatever its scope and definition, is always dual. In fact, we can even go as far as to say that the psychological effects (psychological footprint) of the COVID-19 pandemic will, as in most pandemics, be much higher, more pronounced, more widespread, and longer-lasting than the somatic effects of the infection (medical footprint) (6, 7). The COVID-19 pandemic, as a global experiment, without prior consent nor the approval of any ethics committee, has seriously affected us, continues to affect us, and will affect us throughout the journey of life. No forgiveness for the virus.

The continuous and immense scientific literature on the subject, revealed the direct and indirect, short and long term, effects of COVID-19 on the Central Nervous System (CNS), leading to the decline in people's mental health. In all of these cases, some of the social determinants of mental health, and their impact on disadvantaged populations in times of crisis, can help policymakers establish action plans to mitigate the mental health turmoil of the COVID-19 pandemic, both during this period and in what is to come.

The studies included in this special volume, dedicated to the effects of the COVID-19 pandemic on eating behavior and mental health constitute just a small sample of issues related to food and nutrition. Gathered together, they immediately reveal that the global mental health crisis, especially in relation to lifestyles and eating behaviors (Cachero et al.; Ge et al.; Paz-Graniel et al.), caused by COVID-19 has lasted longer than we all expected. With its high uncertainty and limited control, the COVID-19 pandemic has affected all populations. Of course, the studies summarized here show much more than that, but they also reveal that individual differences exist and are caused not only by personality factors, but also by psychological and social factors, such as economic, educational, health, and social differences, which plague a large proportion of nations around the world.

Considering that eating behavior involves mechanisms that respond to social pressures, the papers in this Topic seek to elucidate how mental health problems during the pandemic, including depression and anxiety, relate to eating behaviors and body image (Wu et al.; Yang et al.). Many among them have examined the correlation between mental health and eating behavior, eating habits, and consumption patterns during the COVID-19 pandemic (Besoro-Moreno et al.; Cachero et al.; Galvão et al.; Ge et al.; Yang et al.).

We are grateful to our countless colleagues and friends for the support and encouragement they have collectively given us. Moreover, we mainly recognize the authors and reviewers for the manuscripts that comprise this special issue with attention and precision. We will be immensely rewarded if this volume helps to advance psychological knowledge about the effects of the COVID-19 pandemic on the behaviors and eating habits of the global population, in the hope that it will assist us in coping adaptively, or resiliently, with future infectious diseases.

## Author contributions

RG: Conceptualization, Writing—original draft, Writing—review & editing. JdS: Conceptualization, Writing—original draft, Writing—review & editing. JS: Conceptualization, Writing—original draft, Writing—review & editing.
